# Outcomes and predictive factors in scleral buckle surgery for rhegmatogenous retinal detachments

**DOI:** 10.1007/s00417-025-07093-0

**Published:** 2026-01-22

**Authors:** Mariam El-Abiary, David Yorston, D Alistair H Laidlaw, Tom Williamson, David H. Steel, BEAVRS/Euretina Outcomes Study Group, Craig Goldsmith, Steven Rowley, Stephen Wnder, Kurt Spiteri Cornish, Ibrahim Masri, Jonathan Smith, Diego Sanchez-Chicharro, Atiq Babar, Kamaljit Singh Balaggan, Marta Figueroa, Julio J. Gonzalez-Lopez, Edward Herbert, William Luke Membrey, Timothy Cochrane, Izabela Mitrut, Laura Wakely, Vasileios Papastavrou, Niall Crosby, Vegard Forsaa, Deepak Vayalambrone, Roxane Hillier, Sandro Di Simplicio Cherubini, Assad Jalil, Stephen J. Charles, Tsveta Ivanova, Abdallah A. Ellabban, Aman Chandra, Imran J. Khan, Paul Y. Chua, Shi Z. Tan, Rumana N. Hussain, Heinrich Heimann, Ian A. Pearce, Teresa Sandinha, Carl Groenewald, Fidan Jmor, Yannick Le Mer, Edward J. Casswell, Tony Casswell, Vaughan Tanner, Angelina Meireles, Niels Crama, John Ellis, Sonali Tarafdar, Huw Jenkins, Andrew Davies

**Affiliations:** 1 Consultant Vitreoretinal Surgeon, Ninewells Hospital, Dundee, Scotland; 2 Consultant VR Surgeon, Tennent Institute of Ophthalmology, Glasgow, Scotland; 3 Consultant VR Surgeon, NHS Foundation Trust, Guy’s & St Thomas, London, UK; 4 Consultant VR Surgeon, Sunderland Eye Infirmary, Sunderland, UK; 5 Bioscience Institute, Newcastle University, Newcastle Upon Tyne, UK

**Keywords:** Retinal detachment, Scleral buckle, Proliferative vitreoretinopathy, Rhegmatogenous, Round hole, Retinal surgery, Macula, Visual acuity, Retinal dialysis

## Abstract

**Background:**

Over the last 25 years, there has been a shift away from *ab externo* scleral buckling to vitrectomy and internal tamponade for the repair of rhegmatogenous retinal detachments (RRD). Despite this, there are still specific indications for scleral buckle. There is little recent research on which patient and surgical factors influence the success or failure of buckle surgery for RRDs.

**Methods:**

A review of 1015 eyes in the BEAVRS/Euretina database treated by a scleral buckle between January 2011 and September 2022. Demographics, characteristics of the RRD, and details of the buckling procedure were assessed to determine which factors were associated with surgical success. Success was defined as an attached retina with a minimum follow up of 6 weeks. Potential risk factors were analysed by multivariable logistic regression.

**Results:**

54.4% of the patients were male, and the median patient age was 37. The single operation success rate was 87.5%. Factors associated with an increased risk of failure include the presence of a U-tear, compared to a round hole or dialysis (OR 3.18, *p* < 0.001), PVR B or C (OR 2.07, *p =* 0.03), involvement of more than one quadrant (OR 2.03, *p =* 0.007), lowest break above midline (OR 1.68, *p =* 0.03), age (OR 1.02 per year, *p =* 0.007). A reduced risk of failure was associated with: surgery performed by a high volume surgeon (defined as ≥ 45 buckle procedures; OR 0.62, *p =* 0.027), the use of a sponge (OR 0.47, *p =* 0.002), macula on detachment (0.61, *p =* 0.03) and performing stab needle drainage (OR 0.41, *p =* 0.001). In patients under 40, with detachments caused by round holes or retinal dialysis, the retina was reattached with a single operation in 91.1% of eyes.

**Conclusions:**

We identified patient and surgical variables which are associated with buckle failure. This study confirms that, scleral buckle procedures still have a role in treating RRD. Sub-retinal fluid drainage, and use of a sponge rather than a silicone tyre, may increase the probability of surgical success.

## Key messages

**What was known:**

- *There is a paucity of data on which patient and surgical factors influence buckle failure.*
- *Previously identified factors associated with failure include macula off status and the presence of PVR.*

**What is new:**

- *We identified that the presence of a horseshoe tear, PVR B or C, macula off status and increasing age are all associated with increased risk of buckle failure.*
- *Factors associated with a reduced risk of failure include an experienced surgeon, the use of a sponge, and performing stab needle drainage*

## Introduction

Over the last 25 years, there has been a significant shift away from *ab externo* scleral buckling to vitrectomy and internal tamponade for repairing rhegmatogenous retinal detachments (RRD) [1, 2].

Despite the change in practice, it is recognised that there are still specific indications for scleral buckling. The Primary Retinal Detachment Outcomes Study [3] reported a 91.7% single surgery success rate for moderate complexity RRDs and it was associated with a better visual outcome compared to combined vitrectomy and scleral buckle. In a recent single centre study from the UK, the primary re-attachment rate was 83.7% [4]. The landmark Scleral buckle versus Primary vitrectomy in Rhegmatogenous retinal detachment (SPR) trial [5], which compared scleral buckle to vitrectomy in medium complexity RRDs, found a primary anatomical success rate of 63.6% in the phakic scleral buckling group. The same study also showed that buckling was associated with better final visual acuity in phakic patients.

There have been few recent studies which have examined the patient and surgical factors which can affect anatomical outcomes in scleral buckle surgery. The Moorfields study identified posterior vitreous detachment and an inexperienced surgeon as risk factors for anatomical failure [4]. The presence of proliferative vitreoretinopathy (PVR) has been reported to be an important risk factor for buckle failure, as well as the extent of the detachment, foveal attachment and vitreous haemorrhage [6–9]. With this study, we aimed to identify the factors associated with surgical success or failure following scleral buckling in a cohort of 1,015 patients in the UK and Europe.

## Methods

Data for this cohort was collected from the BEAVRS/Euretina retinal detachment database, which is compliant with the UK National Retinal Detachment Dataset [10]. The online web application was specifically designed for the collection and analysis of anonymised complexity based primary retinal detachment surgery outcome data. Data is entered prospectively immediately following surgery, and the outcome of primary success or failure appended at final follow up. The web based structured application utilises a drawing tool to systematically classify each retinal detachment based on extent of the detachment, the break type and location, foveal involvement, and the presence and extent of PVR. Operation details include the type of buckle, sub-retinal fluid drainage, and retinopexy.

Data from primary RRDs repaired with a scleral buckle between January 2011 and September 2022 were extracted. Demographics, characteristics of the RRD and details of the procedure were assessed to determine which factors are associated with surgical success, which was defined as an attached retina at a minimum of 6 weeks follow up without additional re-attachment surgery. Eyes that had a combined vitrectomy and buckle were excluded. All visual acuity measurements were converted to logMAR.

All variables were investigated at the univariate level using χ^2^ tests for categorical variables, and t-tests for normally distributed continuous data. A Mann–Whitney test was used for continuous data that was not normally distributed. Any variable with a p value of < 0.20 progressed to multivariate logistic regression with backward selection employed. A p value of < 0.05 was considered significant.

The non-modifiable covariates considered were patient age and sex, laterality, type of break, location of break, extent of detachment, foveal status, presence and grade of PVR and surgeon experience. Modifiable covariates included the type of buckle used and intra-operative drainage of subretinal fluid. All analyses were conducted using R Statistical Software version 4.4.2 (R Core Team (2021). R: A language and environment for statistical computing. R Foundation for Statistical Computing, Vienna, Austria. (URL https://www.R-project.org/.)) We used the finalfit package(Harrison E, Drake T, Pius R (2024). *finalfit: Quickly Create Elegant Regression Results Tables and Plots when Modelling*. R package version 1.0.8, https://github.com/ewenharrison/finalfit.) for multivariable regression.

This study is compliant with the UK’s data protection act, and the Declaration of Helsinki. The database does not contain any data from which patients or contributors may be identified, and internal identification is by a unique alphanumeric code. No institutional board review or ethics committee approval were required, as, according to UK guidelines, this analysis is considered to be a service evaluation.

## Results

During this period, case details of 13,729 eyes undergoing primary retinal detachment surgery were collected. Of those, 1,269 were repaired with a buckle (9.2%). A minimum of 6 weeks follow up data was available for 1,015 (80%). Follow-up of three months or more was available for only 50.7% of eyes.

Males made up 54.4% of the cohort and right eyes made up 51.6%. The median patient age was 37 (IQR 27–50). 990 (97.5%) eyes were phakic. Cryotherapy was used for retinopexy in 922 (90.8%) eyes. Laser was used in 21 (2.1%), usually in combination with cryotherapy. In 72 eyes (7.1%), the retinopexy method was not recorded. Table 1 shows the patient and surgical variables which were assessed and the associated univariable analysis for primary buckle failure. Where a segmental buckle was utilised, the dataset did not specify whether the buckle was circumferential or radial. The overall single operation success rate for this cohort was 87.5%. In patients aged < 40, with round holes, the success rate was 281/306 (91.8%). For retinal dialyses, the success rate was 235/267 (88.0%). However, for patients aged 40 +, with a horseshoe tear, the success rate was 114/150 (76.0%).

**Table 1:** Univariable analysis of factors associated with anatomical success or failure

		**Success (%)**	**Failure (%)**	**p**
**Age**	Mean (SD)	38.4 (14.8)	43.3 (15.7)	**0.001**
**Sex**	Female	431 (48.5)	49 (38.6)	0.045
	Male	457 (51.5)	78 (61.4)	
**Side**	Right	464 (52.3)	62 (48.8)	0.47
	Left	424 (47.7)	65 (51.2)	
**High volume surgeon**	Low volume	434 (48.9)	74 (58.3)	0.059
	High volume	454 (51.1)	53 (41.7)	
**PVR**	PVR absent	834 (93.9)	109 (85.8)	**0.002**
	PVR present	54 (6.1)	18 (14.2)	
**Foveal attachment**	Fovea off	288 (32.6)	57 (45.2)	**0.007**
	Fovea on	596 (67.4)	69 (54.8)	
**RD extent**	1 quadrant	280 (31.5)	26 (20.5)	**0.015**
	> 1 quadrant	608 (68.5)	101 (79.5)	
**Buckle material**	Solid	581 (66.1)	93 (73.8)	0.105
	Sponge	298 (33.9)	33 (26.2)	
**Break type**	Round hole	466 (52.5)	47 (37.0)	** < 0.001**
	Dialysis	235 (26.5)	32 (25.2)	
	Others	34 (3.8)	4 (3.1)	
	U tear	153 (17.2)	44 (34.6)	
**SRF drainage**	None	533 (60.2)	86 (67.7)	**0.048**
	Cutdown	109 (12.3)	19 (15.0)	
	SND	244 (27.5)	22 (17.3)	
**Break position**	Below midline	510 (57.4)	63 (49.6)	0.197
	Midline	134 (15.1)	20 (15.7)	
	Above midline	244 (27.5)	44 (34.6)	
**Encircling buckle**	Segmental buckle	842 (94.8)	125 (98.4)	0.117
	Encirclement	46 (5.2)	2 (1.6)	

Sixty surgeons contributed cases to the dataset. The mean number of cases performed by each contributor was 18.9. Nine surgeons carried out 50% of the operations. All nine surgeons had 40 or more operations in the dataset.

Table 1 shows the univariate analysis. Factors associated with surgical success include the presence of a round hole or dialysis detachment compared to a horseshoe tear, younger age, the use of a sponge compared to a solid tyre, stab needle drainage of subretinal fluid, foveal attachment, ≤ 1 quadrant involvement, the absence of PVR compared to PVR grade B or C, and an experienced surgeon (defined as a user with 40 or more scleral buckle procedures in the database).

There was no association found between anatomical outcome and laterality (*p =* 0.47) or lens status (*p =* 0.258).

**Fig. 1:**
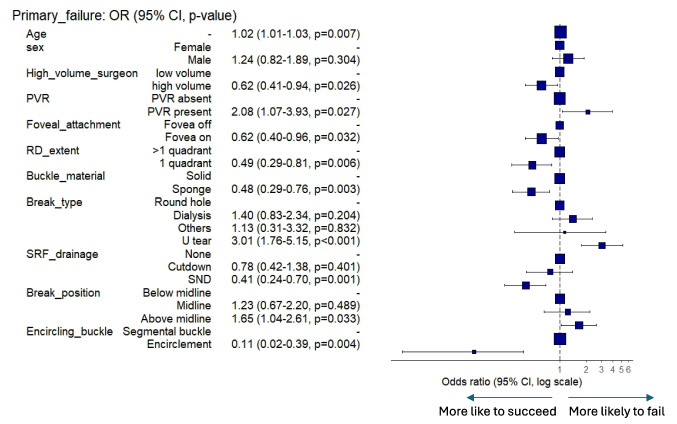
Odds ratios for primary anatomical failure

Table 2 and Fig. 1 show which variables remained significant following multivariate logistic regression. The following factors reduced the risk of failure following scleral buckle surgery: encircling buckle (OR 0.1), stab needle drainage (OR 0.41), macula on at presentation (OR 0.61) sponge buckle material (OR 0.47) and a high-volume surgeon (OR 0.62).

**Table 2:** Multivariable regression analysis of factors associated with anatomical outcome, showing the odds ratio for failure

		**Success (%)**	**Failure (%)**	**O.R**	**O.R. 95% c.i**	**p**
**Age**	Mean (SD)	38.4 (14.8)	43.3 (15.7)	1.02	1.01–1.03	**0.007**
**Sex**	Female	431 (89.8)	49 (10.2)			
	Male	457 (85.4)	78 (14.6)	1.24	0.82–1.89	0.304
**High volume surgeon**	Low volume	434 (85.4)	74 (14.6)	-		
	High volume	454 (89.5)	53 (10.5)	0.62	0.41–0.94	**0.027**
**PVR**	PVR absent	834 (88.4)	109 (11.6)	-		
	PVR present	54 (75.0)	18 (25.0)	2.07	1.06–3.90	**0.028**
**Foveal attachment**	Fovea off	288 (83.5)	57 (16.5)	-		
	Fovea on	596 (89.6)	69 (10.4)	0.61	0.40–0.95	**0.028**
**RD extent**	1 quadrant	280 (91.5)	26 (8.5)	-		
	> 1 quadrant	608 (85.8)	101 (14.2)	2.03	1.23–3.45	**0.007**
**Buckle material**	Solid	581 (86.2)	93 (13.8)	-		
	Sponge	298 (90.0)	33 (10.0)	0.47	0.29–0.75	**0.002**
**Break type**	Round hole	466 (90.8)	47 (9.2)	-		
	Dialysis	235 (88.0)	32 (12.0)	1.47	0.88–2.44	0.137
	Others	34 (89.5)	4 (10.5)	1.16	0.137	0.802
	U tear	153 (77.7)	44 (22.3)	3.18	1.88–5.38	** < 0.001**
**SRF drainage**	None	533 (86.1)	86 (13.9)	-		
	Cutdown	109 (85.2)	19 (14.8)	0.78	0.42–1.37	0.398
	SND	244 (91.7)	22 (8.3)	0.41	0.24–0.69	**0.001**
**Break position**	Below midline	510 (89.0)	63 (11.0)	-		
	Midline	134 (87.0)	20 (13.0)	1.25	0.67–2.22	0.468
	Above midline	244 (84.7)	44 (15.3)	1.68	1.06–2.66	**0.026**
**Encircling buckle**	Segmental buckle	842 (87.1)	125 (12.9)	-		
	Encirclement	46 (95.8)	2 (4.2)	0.10	0.02–0.38	**0.003**

RD treated by non-drain operations were less likely to extend over more than one quadrant (χ^2^ = 33.1, *p* < 0.0001), to be macula off (χ^2^ = 15.9, *p =* 0.0001), or to have breaks above the midline (χ^2^ = 8.04, *p =* 0.018), and were more likely to have a sponge buckle than a solid explant (χ^2^ = 27.4, *p* < 0.0001). There were no differences in the types of break, nor in the proportion treated by a high volume surgeon.

The high volume surgeons were more likely to use a sponge buckle than the low volume surgeons (209/499 vs 122/506, χ^2^ = 35.9, *p* < 0.0001), and were more likely to do a stab needle drain (154/506 vs 112/507, χ^2^ = 9.1, *p =* 0.003). Patients treated by high volume surgeons were slightly older (mean age at surgery 40.4 vs. 37.6, t-test = 2.96 *p =* 0.003). There were no differences between high and low volume surgeons in the type of breaks, the extent of the detachments, or the proportion that were macula off at presentation.

Increasing age (OR 1.02/year), a break above the midline (OR 1.68), More than one quadrant involvement (OR 2.03), PVR grade B or C (OR 2.07) and the presence of a horseshoe tear (OR 3.18) were all associated with an increased risk of failure. The model was moderately accurate, with an area under the curve of 0.734.

430 eyes presented with a macula off retinal detachment. Of those, 288 (70.0%) were successfully reattached and visual acuity data was available for 255 eyes (88.5%). At six weeks, 124 (48.6%) achieved a final visual acuity of 6/12 or better, where the median duration of vision loss was 8 days (95% CI 6–11 days). For those achieving less than 6/12, the median duration was 27 days (95% CI 14–57 days, Mann–Whitney U-test, *p* < 0.0001).

## Discussion

The scleral buckle was first developed by Ernst Custodis in 1949 as a way of shortening the retina, but sparing the sclera [11]. His consecutive series of 515 patients was reported to have a 83.3% success rate. Subsequent publications reported anatomical success rates of 75% to 91% after a single operation [12, 13]. More recently, published single operation success rates for moderate complexity RRDs are as high as 91.7% [3]. The primary success rate from this series is 87.5%. Published series of cases undergoing buckling vary greatly in case complexity and follow up. Strict comparison between series is therefore impossible, however our overall primary success rate is broadly similar to those reported elsewhere which range from 63.6% to 93.7%.

Posterior vitreous detachment (PVD) resulting in a horseshoe tear is the most common cause of RRD [14]. While historically, a localised radial buckle was used to treat a solitary horseshoe tear, the presence of the associated PVD, the ease of performing a vitrectomy, and the very high success rate following vitrectomy has resulted in a significant decline in the use of the scleral buckle in these cases. [15, 16] In our study, multivariate analysis shows that there is a significantly higher risk of buckle failure in a horseshoe tear compared to a round hole or dialysis, suggesting that a buckle should be used with caution as a first line to treat horseshoe breaks.

For retinal dialyses and round hole detachments, surgery is indicated when there is evidence of progression, if the patient is symptomatic, or the macula is affected or threatened [15]. In 2005, Ung et al. examined a series of 110 round hole detachments, with 92% being treated with cryotherapy and scleral buckle [15]. The single operation reattachment rate was 99% in eyes treated by scleral buckle. In our series of 513 round hole detachments, the primary success rate was 90.8%.

In cases of retinal dialysis, the primary anatomical success rate was 88.0%, which is consistent with published literature (87%−95.8%) [17–21]. Other treatment modalities have been described (primary vitrectomy with or without encircling band,^19,20,22^ pneumatic retinopexy [23, 24], and laser retinopexy) however, there have been no prospective studies which have directly compared surgical success. A retrospective case series by Rohowetz et al. examined functional and anatomical outcomes of 60 eyes with retinal dialysis undergoing scleral buckle versus combined vitrectomy and buckle [25]. At 6 months, retinal attachment was comparable in both groups (76.9% vs 77.8%) however visual outcomes were significantly better in the buckle only group, in large part due to the use of oil tamponade and the development of cataracts.

Many surgeons consider scleral buckle to be the procedure of choice for retinal dialyses because of their anterior location, the attached posterior hyaloid face, and the affected patients usually being young and phakic [17]. Despite the increasing popularity of vitrectomy in this context, there is no evidence that it achieves better outcomes than scleral buckling. Given the 88% primary success rate in this study, we recommend that scleral buckle remains the first line treatment for retinal dialyses.

There have been few other studies examining the variables which affect scleral buckle success (Table 3).

**Table 3:** Studies examining the factors which affect scleral buckle failure

Study	No of eyes	Type of study	Primary anatomical success	Factors associated with Scleral Buckle failure
Thelen et al 2012 [7]	N = 4325	Retrospective Review of MUSTARD database of RRDs 1980–2001, Munster, Germany	84%	- Macula off status
Soufi et al 2012 [6]	N = 432	Retrospective Review of RRDs 2001–2009, Morocco	72.7%	- Extent of RD - Preoperative PVR - Worsening of postoperative PVR*
Kobashi et al 2014 [8]	N = 542	Retrospective Review of RRDs in Kanagawa, Japan	93.7%	- Macula off status*
Salabati et al 2023^9^	N = 499	Retrospective Review of RRDs, Philadelphia, USA	86%	- Male gender* - Macula off status* - Preoperative PVR*
Muqit et al. 2024	N = 589	Retrospective review of RD, London, UK	83.7%	- Posterior vitreous detachment* - Inexperienced trainee surgeon*
This study 2024	N = 1015	Review of prospectively collected structured registry data in BEAVRS database of RRDs, 2011–2022, UK/Europe	87.5%	- Horseshoe tear* - Preoperative PVR B or C* - Macula off status* - Break location above midline* - Increasing age*

(*statistically significant on multivariable analysis)

PVR is a recognised risk factor for RRD failure, in both vitrectomy [26] and scleral buckle surgery. In most of the large cohort studies comparing scleral buckle to vitrectomy, [5, 27, 28] such as the SPR trial, or the Primary Retinal Detachment Outcomes Study (PROS), the presence of pre-operative PVR (usually grade B or C) was an exclusion criterion, therefore its effect was not analysed when looking at surgical success rates. In our cohort, the presence of preoperative PVR B or C was associated with an increased risk of failure. This finding has also been reported recently by Salabati et al [9] who found that a scleral buckle was much more likely to fail in the presence of PVR B or C, compared to the absence of PVR (OR 4.27).

In our study, we found that a detached macula at presentation was associated with an increased risk of failure. This is echoed by Salabati et al [9] and by Kobashi et al. [8] The large database review by Thelen et al [7] also reported the chance of anatomical success was 8% lower if the macula was detached compared to attached, however the follow up time frame in this study was very short (5–10 days) and there was little information on baseline characteristics, or surgical technique, and no multivariable analysis.

The extent of the RD is closely linked to macular status and duration of detachment. In our study, 10.9% of presenting RRDs involved ≥ 3 quadrants, whereas the proportion is 20.6% in Salabati et al.’s study. ≥ 3 quadrant involvement was not found to be a risk factor for failure in either study, however in our study, involvement of ≤ 1 quadrant was associated with an increased probability of anatomical success.

The presence of retinal breaks above the horizontal midline was found to be a risk factor for buckle failure in our cohort (OR 1.57). This has not been discussed in the other studies assessing primary buckle failure. Since the database was initially intended for vitrectomy surgery, it automatically calculates the position of the lowest break, as inferior breaks are known to reduce primary success rates in RRD treated by vitrectomy. Where the lowest break is above the midline, we can be confident that a superior break is present. However, if it is below the midline, we cannot exclude additional superior breaks. It is likely that this study underestimates the frequency of superior breaks.

58.7% of the Salabati et al.’s cohort had an injection of gas tamponade (either SF_6_ or C_3_F_8_). Gas tamponade was used in only 10.3% of our series. In eyes with breaks above the midline, the failure rate was 40/239 (16.7%) with no tamponade, compared to 4/49 (8.2%) in eyes with intraocular air or gas (O.R 0.44, 95% c.i.0.15–1.3) Although the numbers are small, and the benefit remains uncertain, it is plausible that an intraocular gas bubble may improve anatomical success rates in scleral buckling, where there are breaks above the horizontal midline.

In our cohort, we found that increasing patient age is a significant risk factor for buckle failure (OR 1.02/year). While this was not found to be a significant factor in the American, Japanese and Moroccan cohorts, it has been described before by Park et al. [29] This retrospective review of 127 patients undergoing scleral buckling for uncomplicated macula off RRDs excluded eyes with previous surgery, including pseudophakia, multiple tears, and PVR C. The authors reported primary buckle success of 77.4% in the cohort aged above 35, and 92.4% in the cohort aged below 35 (*p =* 0.042). Increasing age is associated with a greater likelihood of a posterior vitreous detachment. We did not include vitreous status in our model as it may be difficult to determine by clinical examination alone. The vitreous status was unknown/uncertain in over a quarter of the eyes in our study. The Moorfields scleral buckle study found age was not a significant predictor of outcome, but posterior vitreous detachment was strongly associated with a reduced primary success rate (odds ratio 0.21, 95% c.i. 0.1 to 0.49). The practical implication of this is that, in the occasional detachment patient who presents in their 50’s with an attached vitreous, it is reasonable to do a scleral buckle procedure.

Our data suggests that performing stab needle drainage was associated with an increased probability of primary anatomical success compared to non-drainage, although non-drain surgeries were likely to performed in eyes with a better prognosis. However, sub-retinal fluid drainage is associated with complications such as subretinal haemorrhage and retinal incarceration. Surgeons must decide if the better anatomical outcome justifies the risk of complications in each case [8]. Our data also suggests that solid silicone tyres were less likely to be successful than silicone sponges. We do not have data on buckle orientation, as buckles were classified as either encircling or segmental, with no further classification as radial or circumferential. Both external drainage, and choice of buckle material, require further studies to confirm their effect on buckle success.

Half of the operations were performed by nine surgeons. These “high volume” surgeons had a higher success rate than the other 51 surgeons, and this cannot be entirely explained by the presenting features, nor by the use of sponge buckles and stab needle drainage. Muqit et al [4] showed that surgery performed by an inexperienced trainee was less likely to be successful. As scleral buckling makes up a smaller proportion of retinal surgeries, how can we ensure that future vitreoretinal surgeons can achieve competence in this technique? We recommend that all ophthalmology trainees should be competent using an indirect ophthalmoscope, as this is critical to successful scleral buckling. Secondly, the use of simulation workshops can be helpful in preparing trainees for scleral buckle surgery. The Britain & Eire Association of Vitreoretinal Surgeons (BEAVRS) runs regular scleral buckle workshops [30], which allow trainees and consultants to practise scleral buckle techniques intensively. It is already known that simulation training improves patients outcomes in cataract surgery [31], and it is likely that this would also be true for scleral buckles.

This study has some weaknesses. Strict anonymisation means that there is no patient identifier to use patients as a cluster variable in the modelling; however, we believe that only a small number of patients would have had bilateral RD treated by scleral buckle in the time frame of the data collection. The database was originally designed to collate data from vitrectomies, and so did not include buckle orientation or the position of the most superior break. The latest version of the database does include these variables. Most of the data comes from the UK, where encirclements are uncommon. Visual acuity data was collected, but there were relatively small numbers of macula off detachments, so we cannot draw meaningful conclusions about factors influencing final visual acuity. There are few pseudophakic eyes included, and our findings should only be applied to phakic retinal detachments. Success was defined as an attached retina at six weeks, and we acknowledge that this is a short follow-up period. The median time to failure was 30 days, and 90% of failures occurred within 12 weeks of surgery Ideally, we would have had a minimum follow-up of 12 weeks. However, only 49% of eyes were followed for 12 weeks or longer. The major strength of this study is the large cohort.

Scleral buckles still give high success rates in appropriately selected cases. A recent publication by the BEAVRs database study group [32] provides risk stratification for patients undergoing vitrectomy for RRDs. In the current study, the failure rate for detachment caused by a superior horseshoe tear in a patient aged over 40, treated by scleral buckle, is 15/62 (24.2%). Previous studies (include BEAVRS and PIVOT) indicate that the expected failure rate would be less than 10% for similar eyes treated by vitrectomy. However, our data also show that, in a patient under 40 with an inferior round hole or dialysis, the failure rate is only 8.7% when treated by a scleral buckle.

## Conclusion

In this study, we identified the presence of a horseshoe tear, PVR B or C, macula off status, a retinal break above the midline and age over 40 as independent risk factors for buckle failure. Appropriate patient selection is advised to improve surgical outcomes. Further studies are encouraged to determine whether external drainage and choice of buckle material influence surgical success.
